# Selective Gelation Patterning of Solution-Processed Indium Zinc Oxide Films via Photochemical Treatments

**DOI:** 10.3390/nano15151147

**Published:** 2025-07-24

**Authors:** Seullee Lee, Taehui Kim, Ye-Won Lee, Sooyoung Bae, Seungbeen Kim, Min Woo Oh, Doojae Park, Youngjun Yun, Dongwook Kim, Jin-Hyuk Bae, Jaehoon Park

**Affiliations:** 1 School of Nano Convergence Technology, Hallym University, Chuncheon 24252, Republic of Korea; sul0129@hallym.ac.kr (S.L.); yw330@hallym.ac.kr (Y.-W.L.); qotndud12@hallym.ac.kr (S.B.); kevenkia0624@hallym.ac.kr (S.K.); owdd123@gmail.com (M.W.O.); doojaepark@hallym.ac.kr (D.P.); 2 School of Semiconductor Display Technology, Hallym University, Chuncheon 24252, Republic of Korea; kth7953@hallym.ac.kr (T.K.); youngjun.yun@hallym.ac.kr (Y.Y.); 3 School of Electronic and Electrical Engineering, Kyungpook National University, Daegu 41566, Republic of Korea; 4 School of Electronics Engineering, Kyungpook National University, Daegu 41566, Republic of Korea

**Keywords:** patterning, solution-processed, indium zinc oxide, thin-film transistor

## Abstract

This study presents a photoresist-free patterning method for solution-processed indium zinc oxide (IZO) thin films using two photochemical exposure techniques, namely pulsed ultraviolet (UV) light and UV-ozone, and a plasma-based method using oxygen (O_2_) plasma. Pulsed UV light delivers short, high-intensity flashes of light that induce localised photochemical reactions with minimal thermal damage, whereas UV-ozone enables smooth and uniform surface oxidation through continuous low-pressure UV irradiation combined with in situ ozone generation. By contrast, O_2_ plasma generates ionised oxygen species via radio frequency (RF) discharge, allowing rapid surface activation, although surface damage may occur because of energetic ion bombardment. All three approaches enabled pattern formation without the use of conventional photolithography or chemical developers, and the UV-ozone method produced the most uniform and clearly defined patterns. The patterned IZO films were applied as active layers in bottom-gate top-contact thin-film transistors, all of which exhibited functional operation, with the UV-ozone-patterned devices exhibiting the most favourable electrical performance. This comparative study demonstrates the potential of photochemical and plasma-assisted approaches as eco-friendly and scalable strategies for next-generation IZO patterning in electronic device applications.

## 1. Introduction

Oxide thin-film transistors (TFTs) have become fundamental components in advanced electronic applications such as displays, sensors, memories, and wearable devices owing to their excellent electrical performance, transparency, and mechanical flexibility [1,2,3]. Among oxide semiconductors, solution-processed materials are particularly attractive because they enable low-cost, large-area manufacturing, making them promising for next-generation flexible electronics [4]. Despite these advantages, solution-processed oxide TFTs still face challenges in terms of achieving high electrical performance, stability, and device-to-device uniformity [5,6,7]. This has motivated intense research on improving the material quality and device architecture to unlock their full potential.

One critical aspect of oxide TFT fabrication is the patterning process, which defines the device geometry and electrical isolation. However, although conventional photolithography is precise, it is often incompatible with solution-based processes owing to its complexity, high cost, and use of toxic chemicals that can degrade oxide films [8]. Alternatively, photopatterning techniques that use photochemical or plasma-based activation have emerged as promising approaches [9]. These methods eliminate the need for photoresists and harsh etchants, simplify the process, reduce the environmental impact, and improve the pattern fidelity [10]. Moreover, patterning plays a pivotal role in minimising leakage currents, reducing fringe effects, and enhancing electrical performance and uniformity. Therefore, the development of effective and eco-friendly patterning strategies is crucial for advancing solution-processed oxide TFT technologies towards commercial viability.

Photoresist-free patterning of sol–gel-processed indium zinc oxide (IZO) thin films can be achieved by ultraviolet (UV)-ozone exposure, pulsed UV irradiation, or O_2_ plasma treatment, each of which employs a distinct surface activation mechanism. UV-ozone treatment uses deep-UV light to generate ozone and other reactive oxygen species that oxidise and remove organic ligands from the precursor film, chemically activating the surface via ozone-induced oxidation [11]. Pulsed UV light drives rapid photochemical reactions in the sol–gel layer, inducing the photolysis of the metal–organic precursors and partial condensation of the inorganic network in the exposed regions. By contrast, O_2_ plasma bombards the surface with energetic oxygen ions and radicals, stripping away organic components and oxidising the film through plasma-induced reactions; however, this method can also induce surface roughness or damage. These different activation principles have important implications for pattern selectivity and film integrity. While previous studies have primarily focused on patterning quality, the underlying chemical reactions and interface growth during gelation have not been thoroughly discussed. In particular, the reorientation of precursor species, decomposition of hydroxyl groups, and gelation of intermediate byproducts under different treatments require further investigation.

In this study, we investigated a photoresist-free patterning approach for solution-processed IZO thin films using three different surface activation methods: UV-ozone, pulsed UV, and O_2_ plasma. This study reveals distinct differences in chemical growth, structural densification, and atomic composition of the oxidised film depending on the applied treatment, which are closely tied to pattern fidelity and the resulting TFT performance. We aimed to clarify the relationship between each treatment method and the resulting device performance by systematically comparing the morphological, chemical, and electrical characteristics of the patterned films. This study provides valuable insights into the optimisation of different photochemical and plasma-assisted patterning techniques to improve the performance, uniformity, and scalability of oxide TFTs, ultimately contributing to the advancement of eco-friendly and cost-effective fabrication strategies.

## 2. Materials and Methods

**Figure 1:**
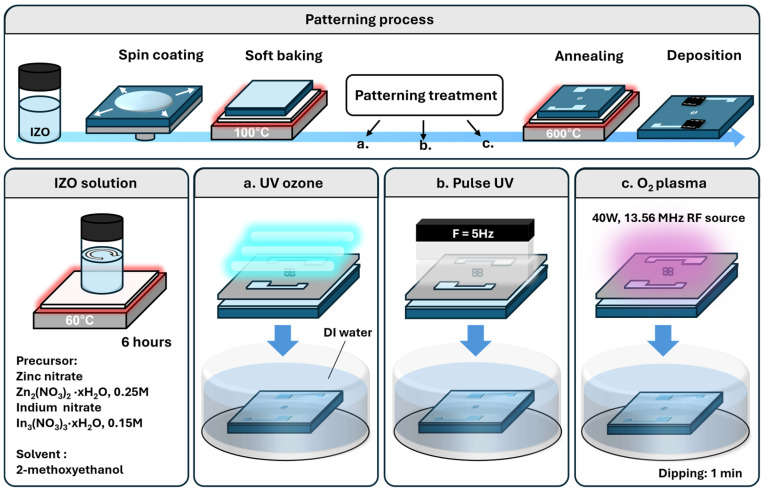
Schematic illustration of the IZO patterning process. The film was treated using three different systems O_2_ plasma, pulsed UV, and UV-ozone and subsequently developed in deionised water to achieve selective patterning.

The overall fabrication process is illustrated in Figure 1. The IZO precursor solution was prepared by dissolving 0.15 M of indium nitrate hydrate (In_3_(NO_3_)_3_·xH_2_O, Sigma-Aldrich, St. Louis, MO, USA) and 0.25 M of zinc nitrate hydrate (Zn(NO_3_)_2_·xH_2_O, Sigma-Aldrich, St. Louis, MO, USA) in 2-methoxyethanol (2-ME). The mixture was stirred at 60 °C for over 6 h until completely dissolved. A 100 nm-thick silicon nitride (SiN_X_) layer was deposited on a p-type silicon wafer via RF sputtering to serve as the gate insulator. The substrates were cleaned by sequential sonication in acetone, isopropyl alcohol, and deionised (DI) water, followed by nitrogen drying and baking at 180 °C for 1 h. O_2_ plasma treatment (CUTE, Femto Science, Hwaseong-si, Republic of Korea) was applied at 40 W for 1 min to enhance the surface wettability. The IZO precursor solution was spin-coated at 3000 rpm for 20 s, followed by soft baking at 100 °C for 10 s. For patterning, the films were selectively exposed to one of three methods through a shadow mask: UV-ozone (UVC-30, Jaesung Engineering Co., Anyang-si, Republic of Korea), pulsed UV (DTX X-UV system, Siheung-si, Republic of Korea), or O_2_ plasma (CUTE, Femto Science, Hwaseong-si, Republic of Korea). Following exposure, the samples were developed in DI water for 1 min to remove unexposed regions. The patterned films were then dried with nitrogen and annealed at 600 °C for 1 h in ambient air. A detailed IZO layer patterning process is presented in Supplementary Materials, Figure S1. Aluminium source and drain electrodes were deposited by thermal evaporation using a shadow mask. For comparison, non-patterned thermal devices were fabricated by spin-coating the IZO solution under the same conditions, followed by soft baking at 120 °C for 5 min and annealing at 600 °C for 1 h. Aluminium contacts were deposited using the same method. The electrical performance of the IZO-based TFTs was measured under ambient conditions using a probe station (Model 4000, MS Tech, Seoul, Republic of Korea). Thermogravimetric analysis (TGA) of the IZO precursor solution was conducted using a TGA N-1000 system (Scinco, Seoul, Republic of Korea) to investigate the thermal decomposition behaviour. Field-emission scanning electron microscopy (FE-SEM) was performed using an SU8600 system (Hitachi, Chiyoda City, Japan) to investigate the surface morphology and cross-sectional structure of the IZO thin films. X-ray photoelectron spectroscopy (XPS) was performed using an NEXSA system (Thermo Fisher Scientific, Waltham, MS, USA) to analyse the chemical bonding states of the IZO films.

## 3. Results and Discussion

We experimentally and empirically demonstrated that the annealing process leading to metal–oxide–metal (M–O–M) oxo-link formation in metal–nitrate precursor solutions proceeds through four major stages: ionisation, gelation, decomposition, and condensation [12,13]. In each state, the IZO precursor solution is densified through the sol and gel states, forming metal–hydroxide–metal (M–OH–M) linkages, which further condense into M–O–M oxo-links. During this sequence, various volatile byproducts, including solvents, nitrous acid (HNO_2_)–water azeotropes, NO_x_ gases, and H_2_O, are sequentially released. The initial ionisation of the metal–nitrate precursor in a 2-ME solvent involves hydrolysis, resulting in the formation of nitric acid and water, as represented by the following reaction:

(1) $$NO_{3}^{-}+3H^{+}+2e^{-}\to HNO_{2}+H_{2}O,\;\;\left(Ionisation\right).$$

During the soft-baking process, thermal energy eliminates most of the volatile components, such as the nitric acid and 2-ME solvent. The IZO precursor transitions from a sol to a gel state, and the reaction can be expressed as

(2) $$M{\left(NO_{3}\right)}_{2}\cdot 3H_{2}O\to M\left(OH\right)\left(NO_{3}\right)\cdot H_{2}O+HNO_{2}↑(gas)+H_{2}O↑\left(gas\right),\;\left(Gelation\right).$$

In this gel state, the metal–nitrate complex contains abundant –OH ligands, which engage in olation reactions to form M–OH–M networks. The nitrate ions within the gel matrix are cleaved and released in conjunction with hydroxyl species. As the temperature increases, nitrate decomposition proceeds, as confirmed in previous studies [8]. The mechanism is summarised as follows:

(3) $$M{\left(OH\right)}_{x}\left({NO}_{3}\right)\cdot H_{2}O\to M{\left(OH\right)}_{x}+{NO}_{x}↑\left(gas\right)+\frac{1}{2}O_{2}↑H_{2}O,\;\;\left(Decomposition\right).$$

Following decomposition, the remaining metal aquo/hydroxy composites undergo condensation, forming stable M–O–M oxo-linkages and releasing water:

(4) $$M{\left(OH\right)}_{2}\to MO+H_{2}O↑\left(Gas\right),\;\;\left(Condensation\right).$$

We propose that, beyond conventional thermal energy, UV-ozone, pulsed UV, and O_2_ plasma treatments can carry out chemical reactions by assisting localised energy. These photochemical or plasma-induced treatments facilitate further decomposition and condensation, enabling selective patterning through negative-tone resist-like behaviour. Because these processes are performed under oxygen-rich conditions, they promote more efficient elimination of gaseous byproducts such as HNO_2_, NO_x_, O_2_, and H_2_O, accelerating the formation of M–OH and M–O network bonds, thereby stabilising the desired pattern.

**Figure 2:**
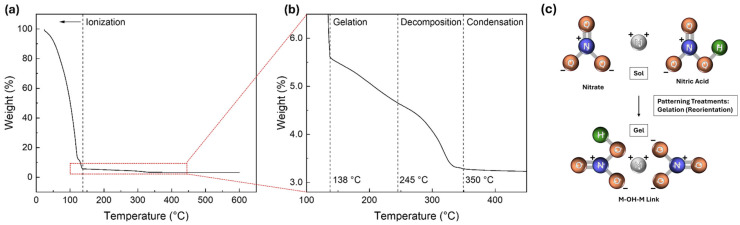
(**a**) TGA curve of the IZO precursor showing overall weight loss up to 600 °C. (**b**) Enlarged view of the marked region in (**a**), highlighting detailed weight loss behaviour. (**c**) Proposed mechanism illustrating molecular reorientation via selective gelation.

The thermal decomposition behaviour of the IZO precursor was first investigated using TGA, as shown in Figure 2, to understand the chemical basis of the patterning process. The TGA curve shows a multi-step weight-loss profile: below approximately 138 °C, precursor ionisation and dehydration occur; between approximately 138 and 245 °C, thermal decomposition of metal–nitrate complexes proceeds with NO_X_ gas release; near 350 °C, residual nitrate decomposition is completed; and above 350 °C, oxolation and condensation reactions lead to the formation of a M–O–M oxide network [13]. These results suggest that surface activation using UV or plasma can locally accelerate the decomposition and condensation reactions [8], triggering reactions that would normally require high thermal energy at much lower temperatures. Consequently, the exposed regions partially develop an insoluble M–O–M framework even before annealing, whereas the unexposed regions retain soluble M–OH species that are easily removed during DI water development [6]. This underlying mechanism establishes the foundation for the photoresist-free patterning strategies analysed in this section.

**Figure 3:**
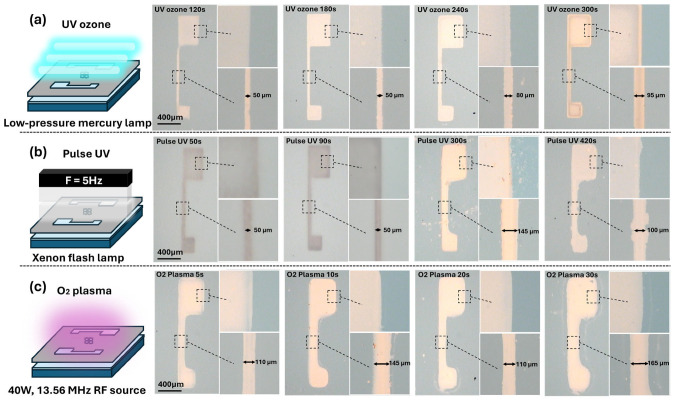
Optical microscope images of IZO patterns formed by (**a**) UV-ozone, (**b**) pulsed UV (F = 5 Hz), and (**c**) O_2_ plasma treatment with different exposure times. Inset images show the wide area (**top**) and narrow line (**bottom**) of each pattern.

Three different exposure methods, namely O_2_ plasma, pulsed UV, and UV-ozone, were employed to pattern the solution-processed IZO thin films, each utilising a distinct surface activation mechanism. Pulsed UV light delivers short, high-intensity light bursts that induce localised photochemical reactions with minimal thermal stress [14], whereas UV-ozone provides continuous and uniform oxidation through low-pressure UV irradiation and in situ ozone generation [15]. By contrast, O_2_ plasma activates the surface via ionised oxygen species generated by RF discharge, allowing for rapid oxidation but with potential surface damage owing to energetic ions [16,17]. Given these fundamental differences in the exposure mechanisms, the resulting film morphology, chemical composition, and device performance were expected to vary depending on the treatment used. Patterned IZO thin films were successfully fabricated using the three photochemical exposure methods in combination with a water-based development process [8]. These approaches demonstrated that patterning is feasible with all three exposure types, as the unexposed regions were selectively removed by DI water following surface modification through photochemical reactions. However, the quality and uniformity of the resulting patterns varied depending on the exposure method used. A clear pattern formation was confirmed for all methods, as demonstrated by the optical microscopy images presented in Figure 3. Additionally, an enlarged image of line pattern and corresponding effective line width as a function of treatment time are presented in Supplementary Materials Figure S2.

Time-dependent pattern evolution was observed for each method. The optical microscopy images for the UV-ozone treatment (Figure 3a) showed progressively enhanced pattern contrast and edge sharpness with an increasing exposure time, indicating gradual and uniform surface activation [18]. In the case of pulsed UV exposure, as shown in Figure 3b, slight improvements in pattern visibility were observed with longer exposures, although no clear or consistent trends were identified. By contrast, the O_2_ plasma-treated films (Figure 3c) exhibited more diffused or broadened pattern edges at longer exposure times, likely because the highly oxidative and energetic plasma environment affected not only the exposed regions but also the masked areas. Additionally, for the O_2_ plasma treatment, no pronounced etching effect on IZO film was observed. Instead, the increase in treatment duration led to an increase in both the effective line width and the observed film thickness. We speculate that this is due to gelation and densification effects rather than material removal. Although differences in pattern definition were observed among the methods, all treatments resulted in increased effective width and film thickness with longer exposure time. Notably, the total process duration required for O_2_ plasma treatment was significantly shorter than for the photochemical methods. In the case of pulsed UV, although the assigned exposure time is longer, the high-peak photon energy is delivered in a millisecond-scale burst at 5 Hz, meaning the actual irradiation time accounts for only ~25% of the total duration. With high-output pulsed UV systems with larger supercapacitors, the effective treatment time can potentially be reduced to 3–30 s compared with our treatment. Based on effective process speed, the methods can be ranked in the following order: O_2_ plasma > pulsed UV > UV-ozone.

**Figure 4:**
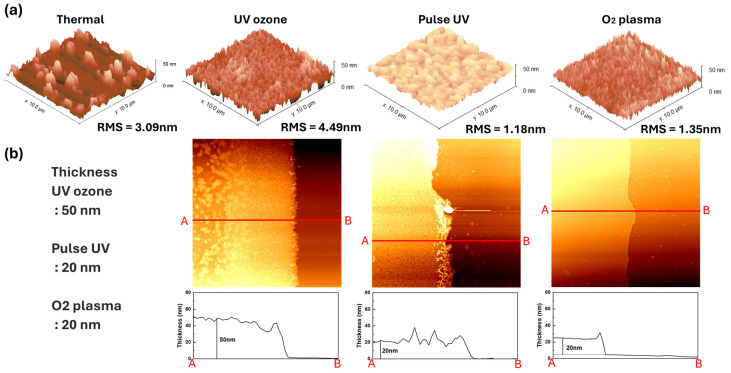
(**a**) 3D AFM images showing the surface morphology and RMS roughness of IZO films fabricated using different treatments: thermal annealing, UV-ozone, Pulse UV, and O_2_ plasma. (**b**) Cross-sectional thickness profiles illustrating the variation in film thickness under different surface treatments. The red lines labeled A and B indicate the positions where the cross-sectional measurements were taken.

Based on the pattern shape alone, the most clearly defined features were observed at 300 s for UV-ozone, 420 s for pulsed UV, and 5 s for O_2_ plasma, as shown in Figure 3a–c, respectively. Atomic force microscopy (AFM) measurements were conducted to investigate the surface characteristics of the patterned films, as shown in Figure 4. Figure 4 shows the morphological and thickness characteristics of the IZO thin films that were subjected to different patterning-related treatments. As shown in the three-dimensional (3D) AFM images (Figure 4a), the surface roughness varied significantly depending on the treatment method. Films treated with pulsed UV and O_2_ plasma exhibited relatively smooth surfaces, with RMS roughness values of 1.18 and 1.35 nm, respectively. By contrast, the films processed by thermal annealing and UV-ozone showed higher surface roughness, indicating similar morphological characteristics, with root mean square (RMS) roughness values of 3.09 and 4.49 nm, respectively. The corresponding cross-sectional profiles (Figure 4b) reveal that most of the films had similar thicknesses (approximately 20–30 nm), except for the UV-ozone-treated film, which was notably thicker (approximately 50 nm). These results suggest that although UV-ozone treatment leads to increased film thickness and roughness, pulsed UV and O_2_ plasma processes are more favourable for achieving uniform and smooth IZO films during the patterning process [19]. From the point of view of patterning, successful pattern formaton was achieved at 300 s of UV-oznone, 420 s of pulsed UV, and only 5 s of O_2_ plasma treatment. Among these, the UV-ozone-treated film exhibited significantly greater thickness, which we attribute to the proloned gelation-enhancing reorientation of precursor and network formation. Additionally, the rms roughness simply appears to increase with the film thickness. The thermally annealed film displayed irregular morphology, likely due to the absence of water development procedure. Although rapid process such as O_2_ plasma and pulsed-UV treatment deliver high power AMD produce thick film within seconds to minutes, their diffusive behaviour over the mask presents disadvantages in terms of pattern fidelity.

**Figure 5:**
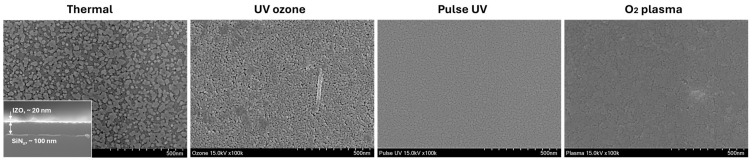
SEM images showing the microstructural differences of IZO films fabricated under various treatment conditions: thermal annealing, UV-ozone, pulsed UV, and O_2_ plasma. The inset in the thermal image presents a cross-sectional view of the film.

Figure 5 presents SEM images of the IZO films processed under different treatment conditions following the AFM analysis, highlighting their distinct microstructural features. The thermally annealed sample exhibited a pronounced granular structure with clearly visible and uniformly distributed crystalline grains. The UV-ozone-treated film also showed a granular morphology but with a rougher and less uniform surface compared with the thermally treated sample, which was consistent with the increased roughness and thickness observed in the AFM and cross-sectional profiles. By contrast, the films treated with pulsed UV and O_2_ plasma exhibited smooth and compact surfaces without prominent grain formation, suggesting the formation of highly uniform thin films [14]. These observations imply that the pulsed UV and O_2_ plasma processes are more favourable for achieving smooth surface morphologies and consistent thicknesses during IZO film fabrication. Eventually, these results align with the idea that prolonged duration may encourage gelation and result in increased roughness. The presence of irregular spots, even within the relatively thin 20–30 nm film formed by thermal annealing, can be attributed to consecutive densification driven by complete oxidation without water rinsing. Based on the surface condition, the film treated with UV-ozone showed a morphology similar to that of the thermal annealed film, suggesting that increased duration of gelation accelerates full oxidation.

**Figure 6:**
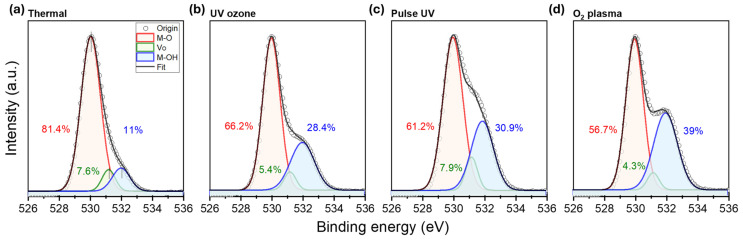
XPS spectra showing the distribution of oxygen-related chemical states in IZO films processed under different treatment conditions: (**a**) Thermal, (**b**) UV-ozone, (**c**) Pulse UV, and (**d**) O_2_ plasma.

XPS analysis was conducted to investigate the chemical compositions of the patterned IZO films further, and the corresponding O 1 s spectra are shown in Figure 6. The peaks were deconvoluted into three component energies corresponding to metal–oxygen bonding (M–O, ~529.0 eV), oxygen-deficient regions often associated with oxygen vacancies (V_O_, commonly centred around ~531.2 eV), and metal–hydroxyl species (M–OH, ~532.0 eV) [17,20]. Notably, the proportion of the M–OH component increased progressively across the treatment types, from 11.0% in the thermally annealed sample to 39.0% in the O_2_ plasma-treated film. This trend may be attributed to both the surface activation effects of photochemical or plasma exposure and the increased hydroxyl adsorption during development in DI water. Although all of the samples underwent high-temperature annealing, the residual M–OH signal suggests incomplete condensation or re-adsorption of the hydroxyl groups that were introduced during the patterning process [19]. While the V_O_ levels remained relatively consistent among all samples, the M–O peak intensity showed a gradual decrease from thermal to plasma treatment. These results indicate that the treatment method significantly influences the surface chemical composition of IZO films, particularly by altering the hydroxylation and M–O bonding characteristics. In particular, the increased coordination between nitric acid and metal centres, rather than the formation of stable metal-nitrate bonding, remaining after selective gelation process, can lead to residual hydroxyl group, as illustrated in the molecular diagram. This irregular metal-hydroxyl bonding was observed more in the results subjected to faster gelation treatments.

**Figure 7:**
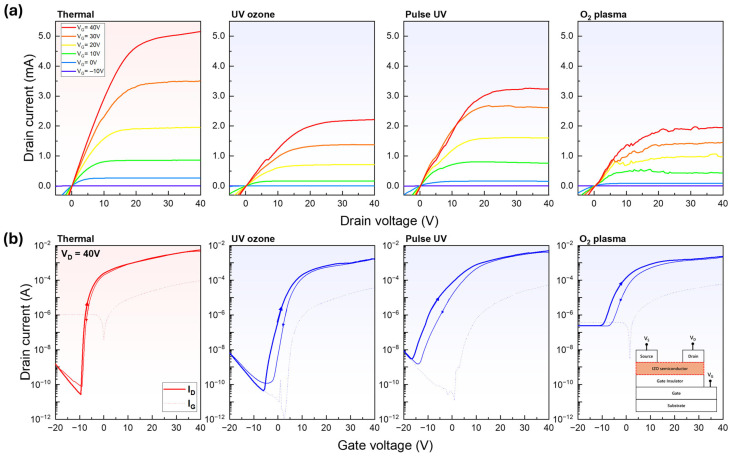
(**a**) Output characteristics and (**b**) transfer characteristics of IZO thin-film transistors fabricated with different surface treatments: thermal, UV-ozone, Pulse UV, and O_2_ plasma.

The electrical output and transfer characteristics of the IZO TFTs fabricated using various surface treatments are illustrated in Figure 7, and the extracted device parameters are summarised in Table 1. Key parameters, including the field-effect mobility (µ), subthreshold voltage swing (S/S), and threshold voltage (Vth), were extracted from the transfer curves in the saturation regimes, V_DS_ = 40 V, to evaluate the electrical performance of the fabricated TFTs. The µ and S/S values were calculated using the saturated drain current of the TFT operation:

(5) $$I_{DS}=\frac{1}{2}\mu C_{ox}\frac{W}{L}{\left(V_{GS}-V_{DS}\right)}^{2}$$

and using the following equation:

(6) $$\mu =\frac{2L}{WC_{ox}}{\left(\frac{d\sqrt{I_{DS}}}{dV_{GS}}\right)}^{2},\;S/S={\left(\frac{d\log I_{DS}}{dV_{GS}}\right)}^{-1}\;.$$

where C_OX_ is the oxide capacitance and W and L are the channel width and length, respectively. The µ value was extracted at the V_GS_ voltage corresponding to the maximum transconductance of the drain current [21]. S/S was determined from the maximum slope of the transfer curve within the subthreshold voltage region [21]. Vth was determined by extrapolating the linear fit of the square root I_DS_ curve. This corresponds to the x-intercept of the tangent line extending at the V_GS_ point where the transconductance is maximised, following the standard for oxide TFTs [22]. The thermally annealed device exhibited the best performance and was used as a reference. To ensure a fair comparison, the patterned devices were fabricated without a photomask, allowing the entire surface to remain active after development and matching the active area of the thermal device. Among the patterned devices moving from the UV-ozone to pulsed UV and O_2_ plasma treatments, the overall channel conductivity increased, as reflected by the higher drain currents and steeper slopes in the transfer curves. However, this improvement in the conductivity was accompanied by a progressive increase in the off-state current, which was particularly pronounced in the plasma-treated device, leading to a degraded S/S and significantly reduced on/off ratios. For example, while the UV-ozone-treated device achieved the highest on/off ratio (3.9 × 10^7^), the O_2_ plasma-treated device showed a drastic reduction to 9.9 × 10^3^, indicating worsened switching performance. These degradations were attributed to the increased M–OH content and decreased M–O bonding, as confirmed by the XPS analysis, which likely facilitated trap-assisted leakage or excess charge transport. To better interpret the device behaviour, the thermally annealed TFT was used as a reference to evaluate the electrical performance of the selective-gelation devices. Compared to this reference, all selective-gelation TFTs showed degraded electrical characteristics, primarily due to unintentional defects during the additional water development process. These electrical degradations were particularly presented in the output characteristics. The superior performance of thermal annealing TFT can be attributed to its well-organised M–O–M oxo-link network, which is formed by sufficient oxygen vacancies and free carriers ionised from indium. In contrast, an increased composition of M–OH groups in the selective gelation introduces inherent H^+^ affinities, which generate excess free electrons. We speculate that these increased carriers result in overall enhancement in conductivity and negative shifts in threshold voltage, as shown in the transfer curves.

**Figure 8:**
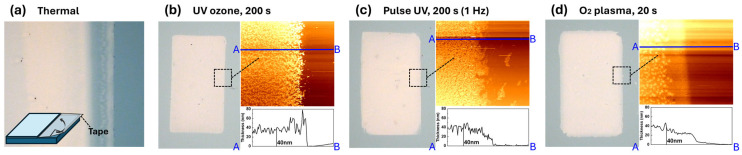
Optical and AFM images of IZO films patterned by (**a**) thermal, (**b**) UV-ozone, (**c**) pulsed UV, and (**d**) O_2_ plasma treatments. All films were fabricated with matched thicknesses for fair comparison. The thermal sample shows a half-coated region formed using tape. The blue lines labeled A and B indicate the positions where the cross-sectional measurements were taken.

The electrical characteristics obtained through photochemical treatments demonstrate that the material properties are effective and suitable for device application; however, there is a need to confirm structural factors such as thickness and patterning. To verify whether the observed differences in electrical characteristics stemmed from variations in film thickness rather than intrinsic material changes, additional patterning experiments were performed under conditions that ensured consistent thickness across all samples. As shown in Figure 8, UV-ozone, pulsed UV, and O_2_ plasma-treatment films were adjusted via exposure time control to achieve a uniform thickness of approximately 40 nm. The AFM line profiles confirmed that the step heights between patterned and non-patterned regions were comparable among the samples. The thermally annealed reference was prepared using a partially masked surface with tape, as it could not be patterned without photochemical treatments. These optimised conditions for uniform thickness were used for fabricating the IZO TFTs.

**Figure 9:**
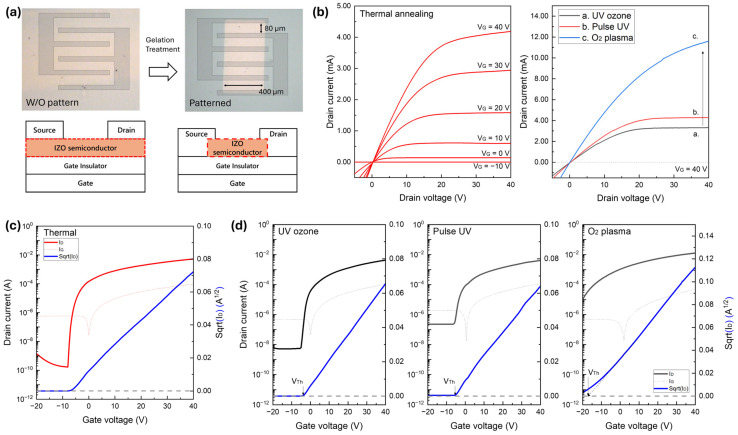
Electrical performance of solution-processed IZO TFTs as a function of patterning treatment. (**a**) Optical image comparing unpatterned (W/O pattern) and patterned IZO films, along with a schematic illustration of the TFT structure. (**b**) Output characteristics of an IZO TFT fabricated with uniform film thickness. (**c**) Transfer characteristics of a solution-processed IZO TFT subjected to thermal annealing. (**d**) Transfer characteristics of patterned IZO TFTs prepared with different gelation treatment conditions.

To investigate the electrical characteristics of patterned solution-processed IZO TFTs with relatively uniform film thickness, the treatment time was carefully optimised to align with the thermal annealing conditions. Despite this optimisation, thermally annealed IZO films could not be fabricated without photoresist-based patterning, necessitating a comparative analysis between patterned and unpatterned devices. As shown in Figure 9, panel (a) presents an optical image comparing the patterned and unpatterned films, along with a schematic illustration of the TFT structure. Figure 9b displays the output characteristics of devices fabricated with thermal annealing and uniform thickness through selective-gelation patterning. Figure 9c depicts the transfer characteristic of thermally annealed TFT and Figure 9d presents the transfer characteristics of patterned IZO TFTs prepared via selective gelation-based treatment methods. The detailed electrical parameters are summarised in Table 2. Although patterned TFTs in uniform thickness showed slightly degraded switching behaviour in off-state current, they still maintained reasonable TFT operation in the relatively same thickness. In consistence with the result in Figure 7, A noticeable trend in conductivity enhancement is observed in the order of UV-ozone, pulsed UV, and O_2_ plasma treatments, which is induced by the ionised free carrier via H^+^ charge in (M–OH)^+^ residue. It is speculated that the limited gelation process caused by rapid exposure duration introduced increased conductivity and instability—a trend more clearly observed in films with uniform thickness. Pulsed UV treatment was found to be less effective than UV-ozone treatment, likely due to insufficient total exposure time for precursor reorientation in gelation. Although O_2_ plasma can assist the gelation in an oxygen-rich atmosphere, it may also promote M–OH formation due to short process time, potentially leading to incomplete M–O bond oxidation. Despite the relatively lower performance of gelation-based patterning compared to thermal annealing, the results demonstrate that, with further optimisation, patterned IZO TFTs can achieve competitive electrical performance suitable for scalable device fabrication.

The subsequent analyses revealed notable differences in the morphology, chemical composition, and electrical characteristics of the IZO films depending on the exposure method. The AFM and SEM results (Figure 4 and Figure 5) showed that the pulsed UV and O_2_ plasma treatments produced smoother and more uniform films, whereas the UV-ozone treatment led to rougher and thicker films despite effective pattern formation. The XPS analysis (Figure 6) indicated a gradual increase in the surface hydroxyl (M–OH) content from the thermal to plasma-treated samples, along with decreased M–O bonding, suggesting reduced film stability. The electrical measurements (Figure 7) confirmed higher conductivity in the pulsed UV and plasma-treated devices but also revealed increased off-state currents, leading to degraded switching behaviour. Overall, although all three exposure methods enabled successful patterning, the UV-ozone treatment offered the best balance between surface quality and device performance, making it a promising approach for solution-processed oxide electronics.

## 4. Conclusions

This study demonstrated a photoresist-free patterning strategy for solution-processed IZO thin films using UV-ozone, pulsed UV, and O_2_ plasma exposure methods, followed by water-based development. Importantly, we closely analysed the selective gelation process by comparing these treatments from the perspective of sol–gel chemistry, focusing on the decomposition mechanism of nitrate-based precursors and the role of the hydroxyl group reorientation in inducing gelation. All three treatments effectively induced selective gelation, enabling patterning without chemical etchants or photolithography. Among them, UV-ozone treatment yielded the clearest patterns and device performance comparable to that of thermal annealing. We speculate that the extended duration or prolonged treatment during the gelation process is key to eliminating residual –OH species and promoting the formation of complete M–O–M oxo-linkages. These findings provide foundational insight into the chemical basis of selective gelation and highlight broader opportunities for tuneable, high-performance metal oxide semiconductor processing.

## Figures and Tables

**Table 1:** Electrical parameters of IZO TFTs by different exposure methods.

Method	$\mu \;({cm}^{2}/V\cdot s)$	Vth (V)	S/S (V/dec)	On/Off Ratio
Thermal	5.8	−5.5	0.4	2.1 × 10^8^ ± 0.1
UV-ozone	3.7	1.3	1.0	3.9 × 10^7^ ± 0.1
Pulse UV	6.1	−3.0	2.3	1.7 × 10^6^ ± 0.1
O_2_ plasma	5.0	−6.1	2.4	9.9 × 10^3^ ± 0.1

**Table 2:** Electrical parameters of IZO TFTs prepared with different gelation treatment conditions.

Method	$\mu \;({cm}^{2}/V\cdot s)$	Vth (V)	S/S (V/dec)	On/Off Ratio
Thermal	4.9	−7.0	0.5	3.1 × 10^7^
UV-ozone	6.7	−4.0	1.3	4.2 × 10^6^
Pulse UV	4.1	−5.4	1.5	1.8 × 10^4^
O_2_ plasma	5.3	−17.1	6.6	1.2 × 10^3^

## Data Availability

The research data presented in this study are available on request from the corresponding author.

## Supplementary Materials

The following supporting information can be downloaded at: https://www.mdpi.com/article/10.3390/nano15151147/s1, Figure S1: Schematic and photographic illustration of solution processed IZO thin film patterning process; Figure S2: Magnified patterning images of the IZO thin film using three different patterning methods.
