# Supplementary material for: Selective Gelation Patterning of Solution-Processed Indium Zinc Oxide Films via Photochemical Treatments
Source: Nanomaterials (Basel). 2025 Jul 24;15(15):1147. doi: 10.3390/nano15151147 (PMC12348702; doi:10.3390/nano15151147)
Supplement: Supplementary file 1 [file nanomaterials-15-01147-s001.zip › nanomaterials-3753410-supplementary.pdf]

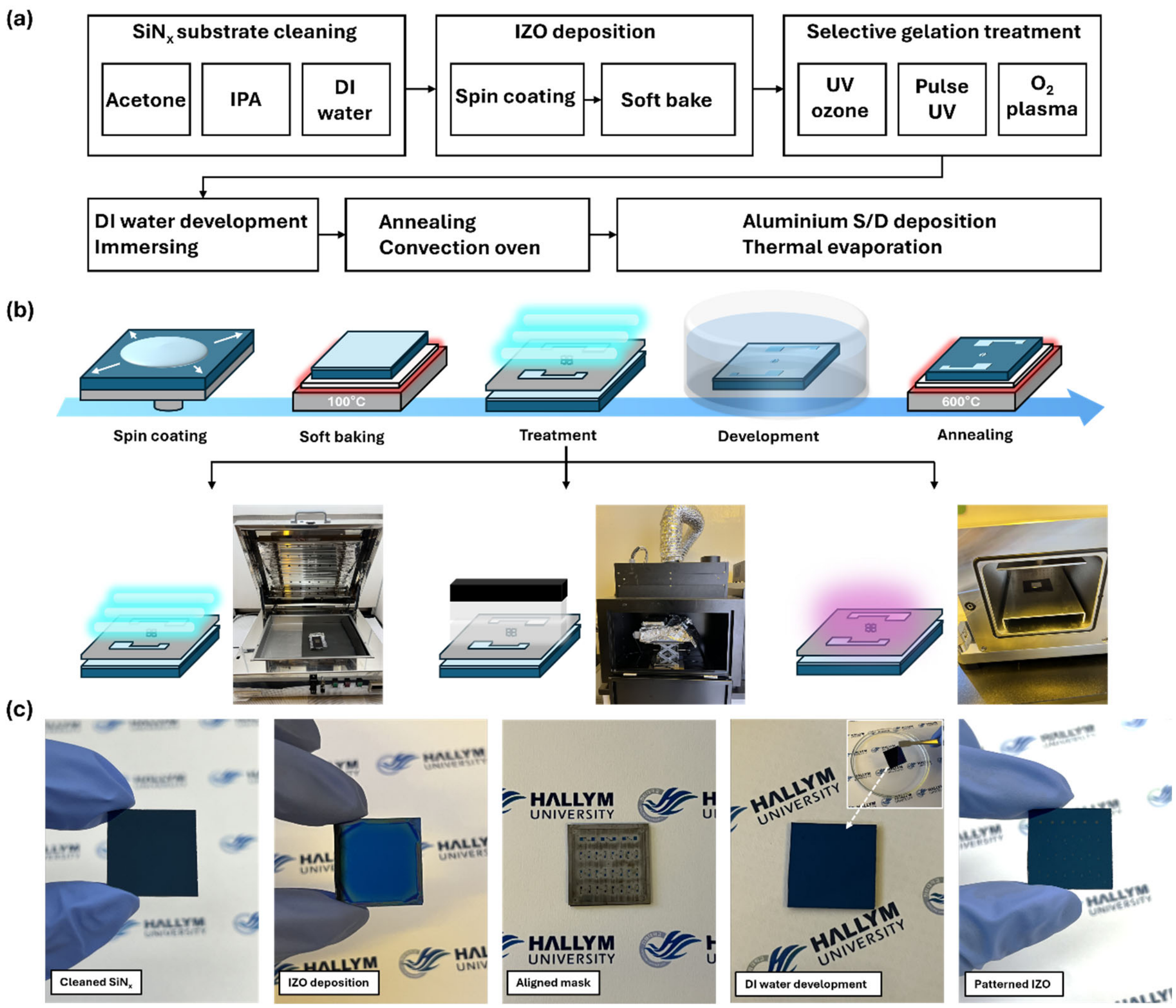

Figsure S1. Schematic and photographic illustration of solution processed IZO thin film patterning process. (a) Process flow diagram for patterning IZO films on SiN_x substrate (b) Photographs showing each key fabrication step (c)Visual schematic and actual equipment images for each step

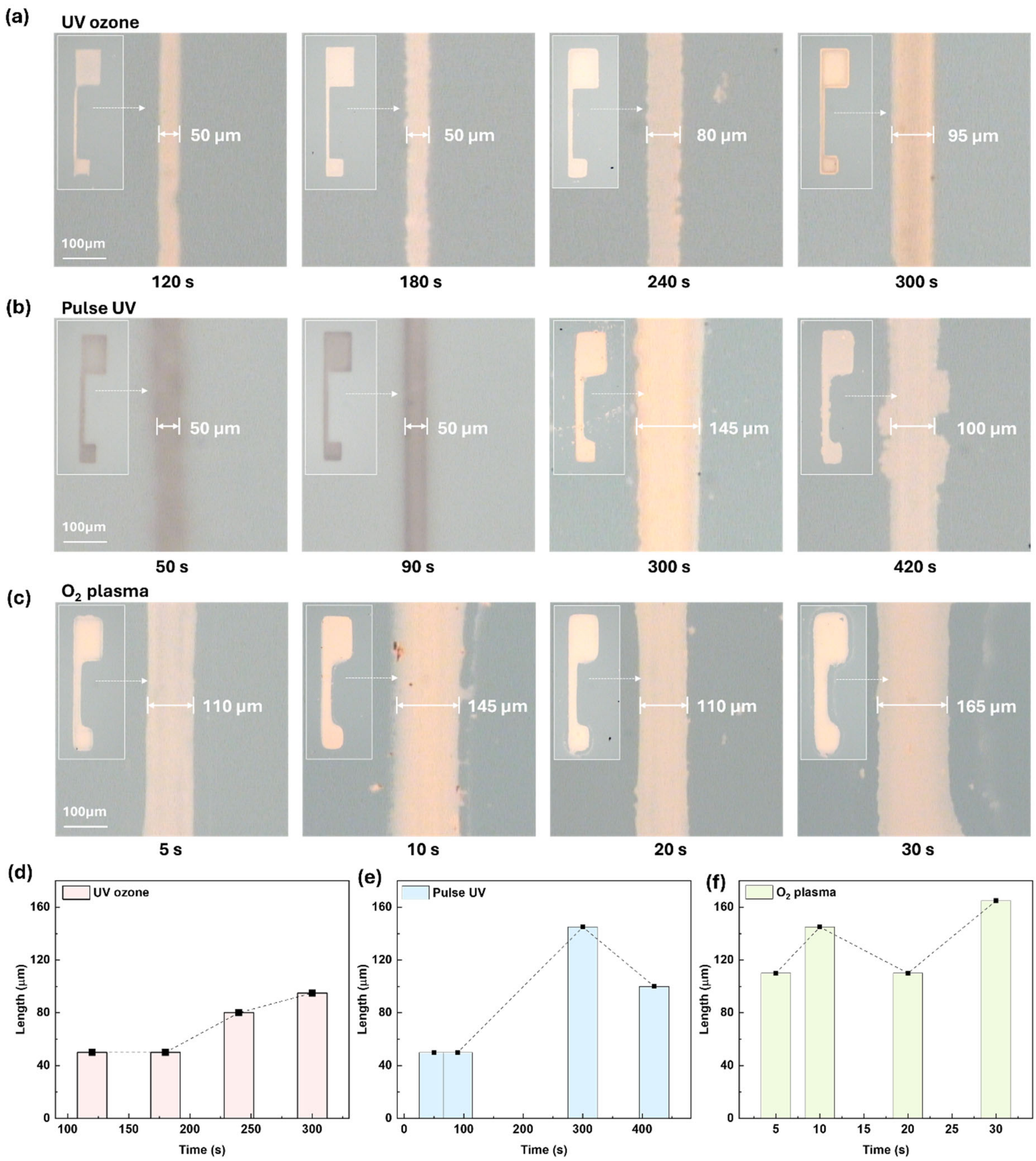

Figure S2. Magnified patterning images of the IZO thin film using three different patterning methods: (a) UV ozone, (b) Pulse UV, and (c) O_2 plasma. The optical microscopy images show the patterned line widths at various treatment times. Plots of pattern widths versus treatment time for each method: (d) UV ozone, (e) Pulse UV, and (f) O_2 plasma.
